# A harmonized database of European forest simulations under climate change

**DOI:** 10.1016/j.dib.2024.110384

**Published:** 2024-04-03

**Authors:** Marc Grünig, Werner Rammer, Katharina Albrich, Frédéric André, Andrey L.D. Augustynczik, Friedrich Bohn, Meike Bouwman, Harald Bugmann, Alessio Collalti, Irina Cristal, Daniela Dalmonech, Miquel De Caceres, Francois De Coligny, Laura Dobor, Christina Dollinger, David I. Forrester, Jordi Garcia-Gonzalo, José Ramón González, Ulrike Hiltner, Tomáš Hlásny, Juha Honkaniemi, Nica Huber, Mathieu Jonard, Anna Maria Jönsson, Fredrik Lagergren, Mats Nieberg, Marco Mina, Frits Mohren, Christine Moos, Xaxier Morin, Bart Muys, Mikko Peltoniemi, Christopher PO Reyer, Ilié Storms, Dominik Thom, Maude Toïgo, Rupert Seidl

**Affiliations:** a TUM School of Life Sciences, Ecosystem Dynamics and Forest Management, Technical University of Munich, Hans-Carl-von-Carlowitz-Platz 2, 85354 Freising, Germany; b Natural Resources Institute Finland, Forest Health and Biodiversity Group, Latokartanonkaari 9, 00790 Helsinki, Finland; c Earth and Life Institute, Université catholique de Louvain, Croix du S, 1348 Ottignies-Louvain-la-Neuve, Belgium; d International Institute for Applied Systems Analysis, Integrated Biosphere Futures Research Group, Schlossplatz 1, A-2361 Laxenburg, Austria; e Helmholtz Centre for Environmental Research UFZ, Permoserstraße 15, 04318 Leipzig, Germany; f Wageningen University & Research, Forest Ecology and Forest Management Group, Droevendaalsesteeg 3a, 6708 PB Wageningen, the Netherlands; g ETH Zürich, Forest Ecology, Institute of Terrestrial Ecosystems, Universitätstrasse 16, 8006 Zürich, Switzerland; h National Research Council of Italy (CNR-ISAFOM), Institute for Agriculture and Forestry Systems in the Mediterranean, Forest Modelling Lab., Via Madonna Alta 128, 06128 Perugia, Italy; i National Biodiversity Future Center (NBFC), Piazza Marina, 61 90133 Palermo, Italy; j Forest Science and Technology Center of Catalonia (CTFC), Crta. de St. Llorenç de Morunys, 25280 Solsona, Spain; k CREAF, E08193 Bellaterra (Cerdanyola del Vallès), Catalonia, Spain; l AMAP, INRAE-CIRAD-CNRS-IRD-Univ Montpellier, 34398 Montpellier cedex 5, France; m Faculty of Forestry and Wood Sciences, Czech University of Life Sciences Prague, 165 21 Prague 6, Kamýcká 129, Czech Republic; n CSIRO Environment, GPO Box 1700, ACT 2601, Australia; o Department of Physical Geography and Ecosystem Science, Lund University, Sölvegatan 12, 223 62 Lund, Sweden; p Potsdam Institute for Climate Impact Research (PIK), Member of the Leibniz Association, Telegrafenberg A 31, Potsdam, Germany; q European Forest Institute, Platz der Vereinten Nationen 7, 53113 Bonn, Germany; r Technische Universität Dresden, Chair of Forest Growth and Woody Biomass Production, Pienner Straße 8, 01737 Tharandt, Germany; s Institute for Alpine Environment, Eurac Research, Via Alessandro Volta, 13A, 39100 Bolzano, BZ, Italy; t Bern University of Applied Sciences, BFH-HAFL, Länggasse 85, 3052 Zollikofen, Switzerland; u Université de Montpellier Université Paul-Valéry Montpellier – EPHE– IRD, CEFE UMR 5175, CNRS, 1919 Route de Mende, F-34293 Montpellier, France; v KU Leuven, Department of Earth and Environmental Sciences, Celestijnenlaan 200E, 3001 Leuven, Belgium; w Swiss Federal Research Institute WSL, Remote Sensing, Zürcherstrasse 111, CH-8903 Birmensdorf, Switzerland; x Université Bordeaux, Bordeaux Sciences Agro, INRAE, Biogeco, 69 route d'Arcachon, F-33612 Cestas, France

**Keywords:** Process-based models, Vegetation dynamics, Europe's forests, Forest development, Forest structure, Forest composition, Forest functioning

## Abstract

Process-based forest models combine biological, physical, and chemical process understanding to simulate forest dynamics as an emergent property of the system. As such, they are valuable tools to investigate the effects of climate change on forest ecosystems. Specifically, they allow testing of hypotheses regarding long-term ecosystem dynamics and provide means to assess the impacts of climate scenarios on future forest development. As a consequence, numerous local-scale simulation studies have been conducted over the past decades to assess the impacts of climate change on forests. These studies apply the best available models tailored to local conditions, parameterized and evaluated by local experts. However, this treasure trove of knowledge on climate change responses remains underexplored to date, as a consistent and harmonized dataset of local model simulations is missing.

Here, our objectives were (i) to compile existing local simulations on forest development under climate change in Europe in a common database, (ii) to harmonize them to a common suite of output variables, and (iii) to provide a standardized vector of auxiliary environmental variables for each simulated location to aid subsequent investigations. Our dataset of European stand- and landscape-level forest simulations contains over 1.1 million simulation runs representing 135 million simulation years for more than 13,000 unique locations spread across Europe. The data were harmonized to consistently describe forest development in terms of stand structure (dominant height), composition (dominant species, admixed species), and functioning (leaf area index). Auxiliary variables provided include consistent daily climate information (temperature, precipitation, radiation, vapor pressure deficit) as well as information on local site conditions (soil depth, soil physical properties, soil water holding capacity, plant-available nitrogen). The present dataset facilitates analyses across models and locations, with the aim to better harness the valuable information contained in local simulations for large-scale policy support, and for fostering a deeper understanding of the effects of climate change on forest ecosystems in Europe.

Specifications Table

**Table:** 

Subject	Environmental Sciences: Ecological modeling
Specific subject area	Harmonizing forest modeling simulations of climate change effects, process-based forest simulation models
Data format	Raw, harmonized
Type of data	Table, Database
Data collection	Data were contributed from the European forest modeling community. Each contributor uploaded simulation data files and a metadata file containing information on the design and drivers of the simulation as CSV files to an R Shiny application. Upon submission, the data were stored in a designated folder with a unique identifier assigned to each contributor. Contributions were required to follow specific criteria, including process-based simulations and annual output information on key vegetation development indicators (i.e. proportion of tree species, canopy height, and leaf area index). Other requirements were that simulation outputs were on the level of tree species, as well as in the absence of disturbances and management (or business-as-usual management).
Data source location	Technical University of Munich, TUM School of Life Sciences, Ecosystem Dynamics and Forest Management Group
Data accessibility	Repository name: *Data for: A harmonized database of European forest simulations under climate change* Data identification number: 10.5281/zenodo.10730807 Direct URL to data: https://zenodo.org/records/10730807

## 1 Value of the Data

• Forest simulation model outputs from 17 different models were collected and harmonized. The dataset contains 1.1 million individual simulation runs over 135 million simulation years across 13,599 unique locations in Europe, covering large proportions of the climate and soil conditions in Europe's forests.
• The database contains standardized output variables across all models. Specifically, harmonized simulation outputs are available for canopy height (structure), leaf area index (LAI; functioning) and tree species proportions (composition) at annual time step. To provide harmonized layers of context information for simulation results, we collated daily climate data for historic climate (1981–2005) and a set of climate change scenarios (2006–2100) and consistent soil proprieties for all simulations.
• This is the first harmonized dataset of local forest model simulations at continental scale. The data collected here will support synthetic analyses of climate change impact on Europe's forests, and will facilitate comparative analyses across locations and models. Further, our dataset also helps to identify regions that remain underrepresented in model-based climate impact assessments and should thus be the focus of future studies.

## 2 Background

The objective was to collate projections on forest development under climate change derived from simulation models. Specifically, we compiled existing simulation data from previously conducted analyses using published models at the stand- to landscape-scale. Contributions to the dataset were made by several experts of the European forest modeling community, and all contributors are co-authors of this paper. Model outputs were compiled for three common state variables describing complementary aspects of forest ecosystems. By harmonizing the output variables across different models and adding standardized climate and soil data we created a novel, bottom-up dataset for broad-scale, multi-model assessments of climate change impacts of Europe's forests.

## 3 Data Description

The data are collected and stored in SQLite format. SQLite is a widely used open-source database format and can be accessed from all major data analysis platforms. One SQLite database contains the raw simulation outputs and a metadata table of all simulations including information about locations and harmonized soil conditions for those locations. This data follows the structure described in detail in the supplementary information (Tables S1 and S2). Simulation outputs with harmonized climate data are stored in one SQLite database per climate scenario. Tables in those databases follow the structure shown in Table 1. Further, a metadata table of all simulations, including information about locations and soil conditions for those locations is provided (Table S2).

**Table 1:** Structure of the harmonized simulation database including standardized auxiliary data for all simulations. Discrete vegetation states were created by combining the three state variables and binning the respective continuous variables – see methods for details. Note that each row in Table 1 describes a column in the simulation database.

Section	Column name	Description
General	SourceID	ID to identify the source of the data (i.e. contributor).
	SimulationID	Unique numeric identifier of the simulation created by the contributor.
	Year	Year of the simulation starting with 1 or the calendar year (e.g., 2000).
Vegetation	Discrete vegetation state	Discrete state derived by combining the three state variables species composition, LAI class and dominant canopy height class (e.g. PIAB_3_20_22; see methods for details).
	Dominant height	In meters, harmonized (calculated from min, mean and max heights, details on the calculations are shown in the methods).
	LAI	Leaf Area Index (one-sided or projected) in m²/m².
Soil	WHC	Water holding capacity of the site (mm).
	TextureSand	% sand content of the soil.
	TextureSilt	% silt content of the soil.
	TextureClay	% clay content of the soil.
	SoilDepth	Depth of the plant-accessible soil (mm) without rocks (> 2 mm diameter).
	AvailableNitrogen	Plant available nitrogen (kg/ha/year).
Climate	Scenario	Combination of GCM and RCP from which daily data was obtained.
	Temperature	Columns “tas_1” to “tas_365” with daily mean temperature [°C].
	Precipitation	Columns “prec_1” to “prec_365” with daily precipitation [mm].
	Radiation	Columns “rad_1” to “rad_365” with daily radiation [W/m^2^].
	Vapor pressure deficit (VPD)	Columns “vpd_1” to “vpd_365” with daily vapor pressure deficit [kPa].

The database contains 1,117,453 simulation runs that together contain 135,375,583 simulation years. Simulations cover 13,599 unique locations across Europe and represent 92 tree species. Simulation data were provided from 19 research groups, using 17 different forest models. All simulations were created with locally tested and evaluated models that are well-documented and published in the peer-reviewed literature (Table 2). Note that as models are further developed over time, model versions used for the simulations may vary from the cited references in some cases. While all models contributed to the coverage of climate and soil conditions across Europe, their individual contributions varied in terms of geographic range and number of simulations provided. Likewise, for some models more data were available than for others. While the majority of simulations were run with iLand, MEDFATE simulations covered the largest climate and soil gradient. The simulations in the database consist of 90.5% climate change runs and 9.5% of the simulations were run under baseline or observed climate conditions. The database contains simulations from 12 stand-level models and four landscape-level models (Table 2, see Bugmann & Seidl, 2022 [1] for a review on modeling approaches). For balance between stand- and landscape-level simulations, a subset of 1-ha stands from the full landscape was sampled and used as individual simulation runs.

**Table 2:** Models, number of simulations per model and their coverage of current climate and soil space of Europe. Percentage of climate and soil space refers to the area covered by the climate and soil space in which the model simulations are located. For this, we stratified the climate and soil space and checked which of the classes are covered by the simulations of each model. We then calculated the area covered by the classes that are represented by each model. Further details are described in the Experimental Design, Materials and Methods section. The Model type column distinguishes between stand-level (S) models and landscape-level (L) models.

	MODEL OVERVIEW					
MODEL	Simulations	% of all	% clim	% soil	Model type	Model reference
ILAND	821,979	73.2	21.4	43.1	L	Seidl et al. 2012 [2]
4C	250,979	22.4	18.3	54.3	S	Lasch-Born et al., 2020 [3]
MEDFATE	22,464	2.0	40.8	64.5	S	De Cáceres et al., 2021 [4]
FORCLIM	9210	0.8	10.3	46.7	S	Bugmann, 1996 [5]; Huber et al., 2021 [6]
LPJ-GUESS2.1	6156	0.6	15.5	46.6	L	Smith et al., 2008 [7]
FORCEEPS	5040	0.5	4.8	6.7	S	Morin et al., 2021 [8]
TREEMIG	2820	0.3	0.6	0.3	L	Lischke et al., 2006 [9]
3PGN-BW	1428	0.1	40.0	59.8	S	Augustynczik and Yousefpour, 2021 [10]
FORMIND	1008	0.1	24.2	6.0	S	Fischer et al., 2016 [11]
3D-CMCC-FEM	367	< 0.1	13.1	7.3	S	Collalti et al., 2018 [12]; Dalmonech et al., 2022 [13]
GOTILWA+	336	< 0.1	10.1	7.2	S	Nadal-Sala et al., 2013 [14]
SORTIE-ND	278	< 0.1	21.3	22.0	S	Canham et al., 2005 [15]
PREBAS	252	< 0.1	6.6	6.2	S	Minunno et al., 2019 [16]
3PGMIX	173	< 0.1	10.1	34.0	S	Forrester and Tang, 2016 [17]
LANDSCAPEDNDC	38	< 0.1	11.3	7.2	L	Haas et al., 2013 [18]
HETEROFOR	12	< 0.1	6.7	18.6	S	Jonard et al., 2020 [19]

**Fig. 1:**
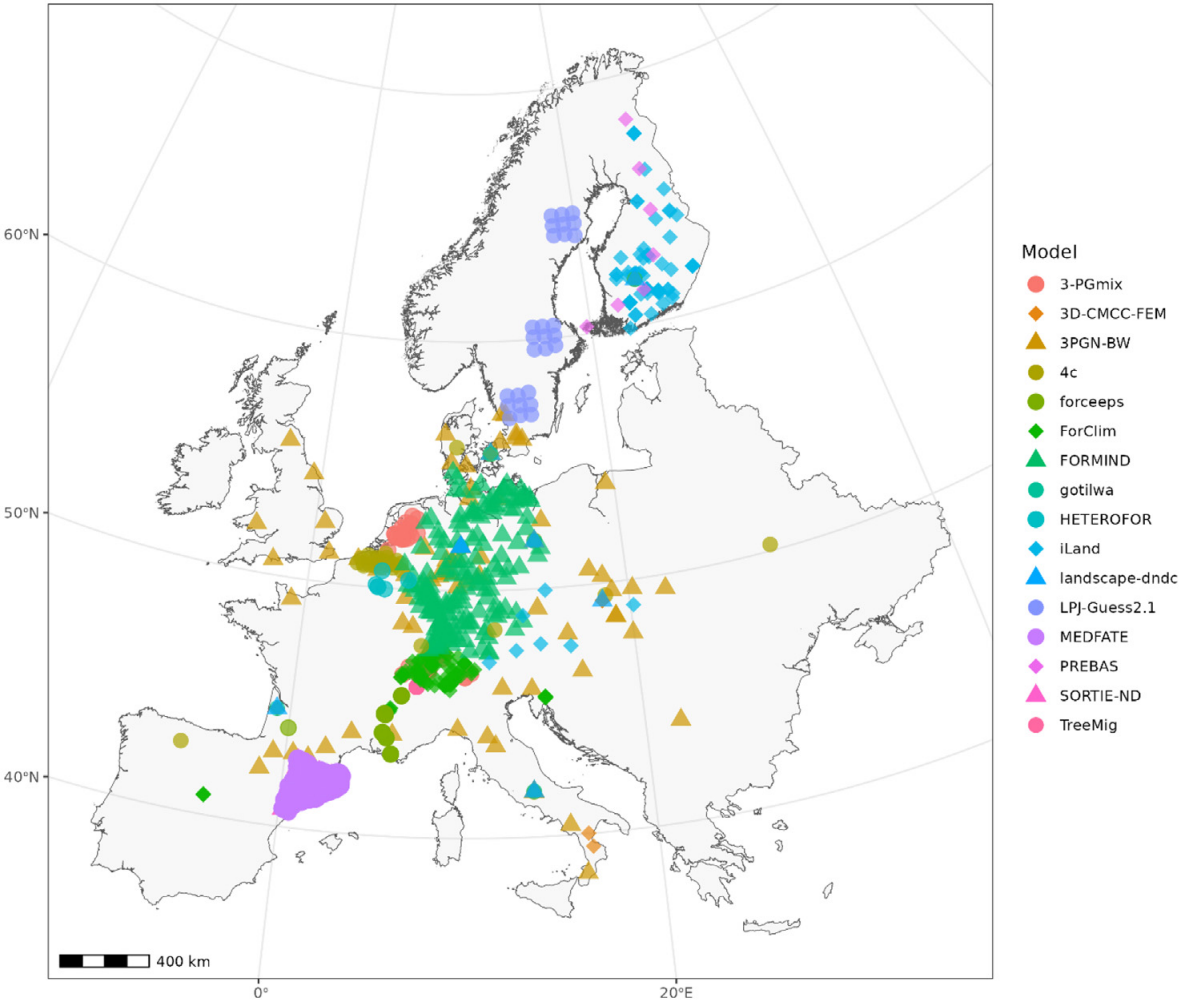
Locations of the simulations in geographical space. Simulations from different models are displayed with different colors and symbols. Note that the projection of the map is Lambert Azimuthal Equal-Area (LAEA), the true north can be identified by following the longitudinal lines plotted in grey at 0° and 20° East.

**Fig. 2:**
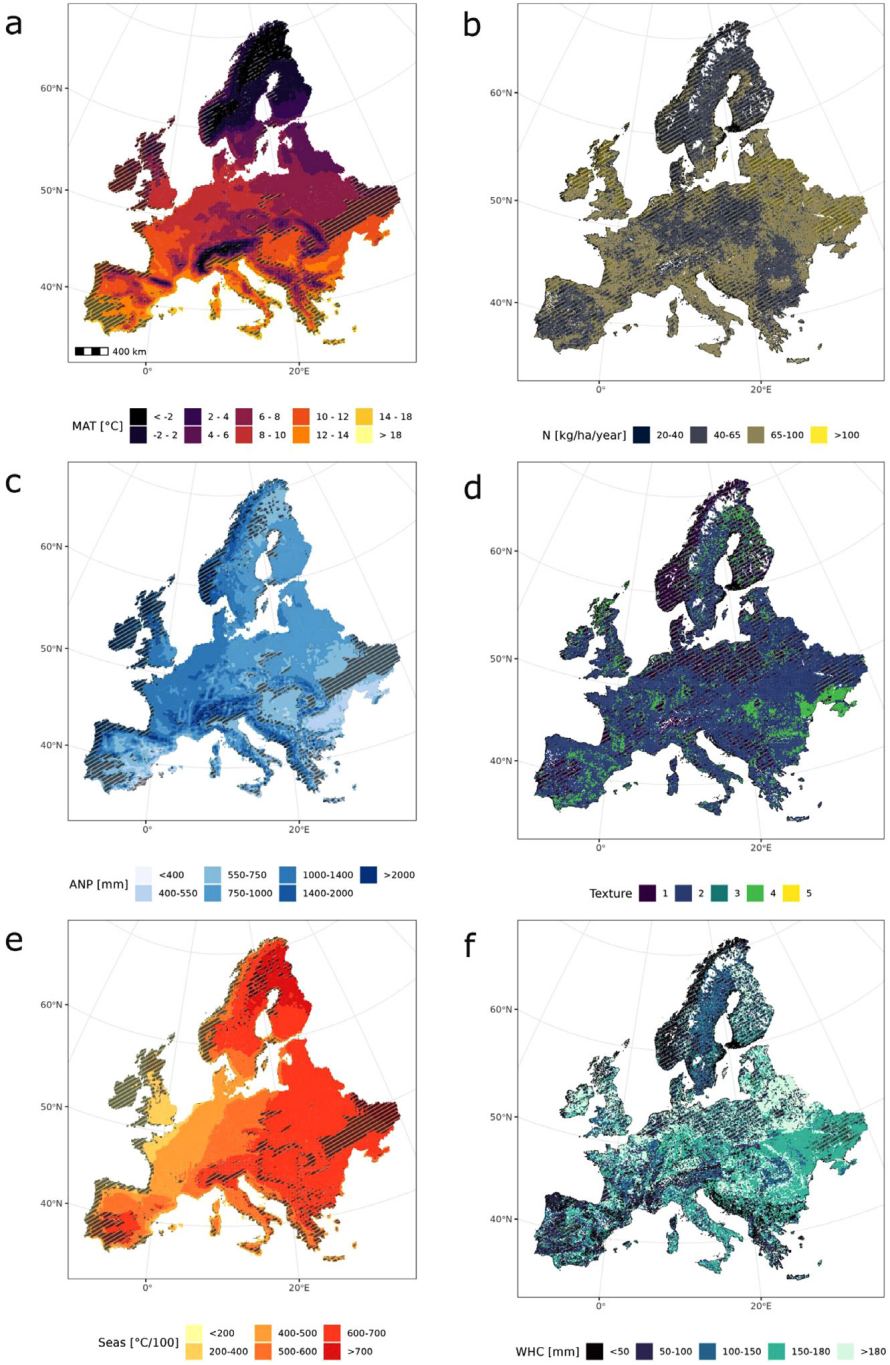
Climate and soil space covered by simulations. Climate space was stratified into unique combinations of mean annual temperature (MAT; ten classes; panel a), annual precipitation (ANP; 7 classes; panel c) and temperature seasonality (Seas; seven classes; panel e). Soil space was stratified to unique combinations of plant-available nitrogen (N; five classes, but 0–20 kg/ha/year is not covered; panel b), soil texture (classes from coarse (=1) to very fine (=5) according to the European Soil Database classification scheme for soil texture; panel d) and water holding capacity (WHC; five classes; panel f). Areas not covered by the data in this database are hatched. For more details and data sources see section 3.2.1 Climate and 3.2.2 Soil.

In geographic space, the simulation runs cluster in Central Europe, Spain (Catalonia), Finland and Sweden, i.e. areas that were analyzed particularly intensively in previous modeling studies (Fig. 1). To evaluate the proportion of the geographic area of Europe that was covered by the climate and soil conditions represented in the dataset, the climate and soil space (using the variables shown in Fig. 2) of the entire continent were stratified and strata in which simulations were located were obtained. As some of the strata cover more geographic area (i.e. climate and soil conditions that occur more often), a simulation in that strata could cover a larger percentage of climate and soil space than other simulations. The climate space covered by all simulation data spans 79% of Europe's geographic area and the covered soil space spanned 75.4% of the geographic area (Fig. 2). Areas not covered are mostly unforested regions in highly continental parts of eastern Europe (i.e. parts of Ukraine, western Romania), very warm regions in the Mediterranean (i.e. southern Spain, parts of Greece and Italy), and wet and very oceanic regions including large parts of the British Isles and southern Norway. For soil conditions, we find that mainly soils with very coarse or very fine texture, low nitrogen availability and low water holding capacity are not covered by simulations.

**Fig. 3:**
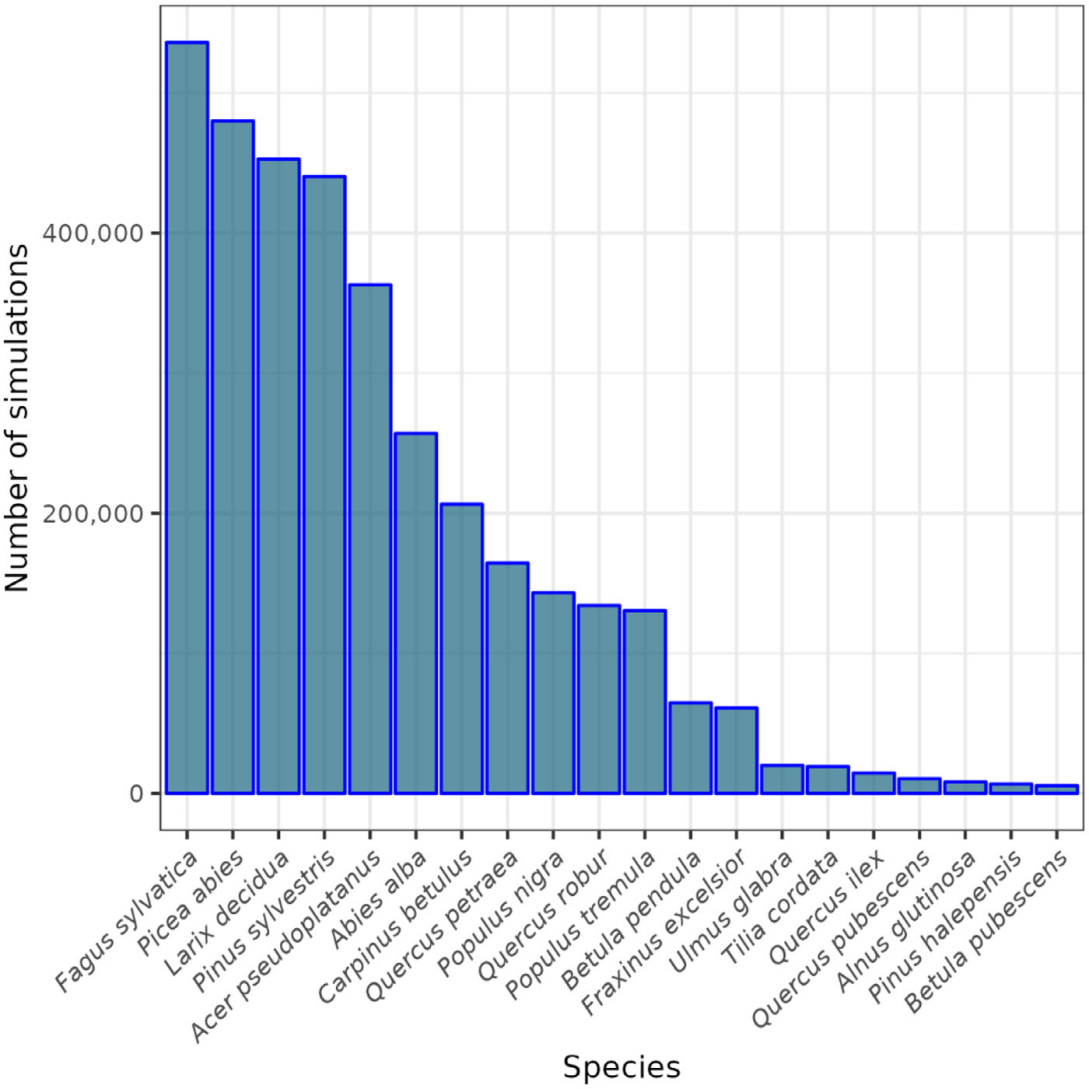
Number of simulations in which the 20 most prevalent tree species in the dataset occur.

The database contains simulations for 92 species. While most species are represented in less than 1000 simulations (median 728), *Fagus sylvatica, Picea abies, Larix decidua*, and *Pinus sylvestris* are the most prevalent, each occurring in over 400,000 simulations (Fig. 3). Furthermore, the dataset contains simulations without forest management (39.2%) and simulations implementing common practices (60.8%).

## 4 Experimental Design, Materials and Methods

### 4.1 Data collection

A central database of forest simulations was created from existing model outputs from previous simulation studies, collating information from a variety of stand- to landscape-scale forest simulation models across Europe. The focus lay on models that were locally evaluated, thus encapsulating the best available bottom-up understanding of quantitative forest ecosystem dynamics in Europe. Furthermore, the dataset was restricted to results from process-based models, as these are expected to be more robust under changing environmental conditions compared to purely empirical models [20]. Simulation outputs were provided by modeling groups in two files, a simulation data file and a metadata file containing information on the design and drivers of the simulation (Table S1 & S2). Forest simulations under climate change conditions were of particular interest, but simulation runs under baseline climate conditions were also included. The minimum requirements for contribution to the dataset were that the model conducting the simulation was process-based, and that simulation outputs provide annual information on basic indicators of vegetation development (i.e. proportion of tree species, canopy height, and leaf area index). While some models provide a broader range of output indicators, these three variables were chosen as least common denominator for describing forest composition, structure, and functioning. Furthermore, the simulations had to provide information at the level of individual tree species, models simulating plant functional types were not included. With regard to the initial conditions of the simulation, both generic initial conditions (such as simulations starting from bare ground) and runs initialized with the current state of the vegetation were included. Further, simulation runs were conducted either in the absence of management interventions or assumed business-as-usual management for the area under study. Natural disturbances were not considered in the simulations. Simulation data were compiled and analyzed at stand level, hence information from landscape-scale simulation models were considered as unique data vectors at the level of simulated stands (i.e., areas of homogeneous climate and soil conditions) within a landscape. The temporal extent of simulations was variable, and both historical and future time series were included.

### 4.2 Data harmonization

Simulation data went through initial checks to ensure that the metadata for each simulation were complete and IDs in the metadata table (Table S2) were matching the simulation data (Table S1). To harmonize simulation outputs across the different models and simulation studies we used discrete vegetation states as described by Rammer & Seidl (2019) [21]. Discrete vegetation states condense the complexity of forest vegetation by describing the structure, composition, and function of vegetation as distinct states. To that end, continuous variables were discretized, and a state was derived for every simulation year in the output of each model. With regard to stand composition, a species was categorized as dominant if it held more than 66% of a standʼs basal area. Admixed species were explicitly considered if their share was ≥20%. Based on these categories, a unique string was derived to describe species composition by concatenating the first two characters of the genus name and the first two characters of the species name (e.g. *Pinus sylvestris* to PISY), and combining all dominant and admixed species occurring in a stand. Letters for dominant species were capitalized, while admixed species were in lowercase letters (e.g. PISYfasy means *Pinus sylvestris* is the dominant species and has a share of more than 66%, with *Fagus sylvatica* as an admixed species with more than 20% of the basal area). To categorize ecosystem functioning, LAI (as a key indicator for the exchange of carbon, water, and energy of the system with the atmosphere) was grouped into three classes: class 1 for sparse (LAI < 2), class 2 for moderate (2 ≤ LAI ≤ 4), and class 3 for dense forest canopies (LAI > 4). For structure, the dominant height of the canopy was employed, as the vertical utilization of space is a key element of forest structure. Canopy height can further indicate the development stage of forest stands. The height information from different models (mean, minimum, maximum) was harmonized to dominant stand height. For stands dominated by *Pinus sylvestris, Abies alba, Larix decidua, Picea abies, Fagus sylvatica* and *Quercus robur*, allometric factors calibrated by Kahn (1994) [22] were applied to describe the relationship of stand dominant height to maximum tree height. For other species, a statistical model based on yield table data was derived to estimate dominant height from mean and maximum tree height [22]. Specifically, for 14 species, a linear mixed model with dominant height as dependent variable, mean and maximum height as predictor variable, and random slopes for the different species was calibrated (conditional R^2^ = 0.999; marginal R^2^ = 0.996). To make the analysis robust to individual outliers, dominant height was limited to lie between 0.8 times maximum height (lower limit) and maximum height (upper limit). Some models only provided information for mean height (but not maximum height), or for minimum and maximum height only (but not mean height). In the latter case, mean height was calculated as the arithmetic mean of minimum and maximum height. For simulations for which only mean height was available, maximum height was estimated using a linear mixed model. This model was calibrated on a random subsample of the dataset of all simulation data across all models that contained both maximum and mean height. In the linear mixed model, maximum height was used as the dependent variable, mean height as predictor variable and random effects for species were included (conditional R^2^ = 0.54; marginal R^2^ = 0.25). Maximum height was then predicted from mean heights with this model and subsequently calculated dominant height from mean and maximum height as described before. Subsequently, continuous information on dominant height was grouped into 2-m bins. Finally, the individual states for forest composition, structure, and function were combined into a unique string describing the state of the vegetation (e.g., PIABfasy_3_20_22 representing a stand dominated by *Picea abies* (PIAB) with admixed *Fagus sylvatica* (fasy) that has a dense forest canopy (LAI class 3) and a dominant height of between 20 and 22 m). This harmonization and discretization of the underlying simulation data resulted in a total of 18,598 distinct vegetation states being recorded in our database.

### 4.3 Auxiliary data

In addition to the harmonized simulation data and the respective metadata, a common data vector of auxiliary data was compiled. This vector contained standardized climate and soil data for each simulation, in order to facilitate the analysis and interpretation of the dataset.

#### 4.3.1 Climate

To derive common and coherent climate data for all simulation runs included in the database, the simulation-specific climate time series (which was restricted to annual values for temperature and precipitation) provided by the modeling groups was matched with climate data from a common climate dataset. This approach allowed to address problems of different data resolution and representation in the data used for individual simulations (e.g., some models operate with monthly climate data while others use daily climate data, some models use maximum and minimum temperature as drivers while others use mean temperature, some models include climatic drivers beyond temperature and precipitation such as vapor pressure deficit (VPD) while others do not, some simulation runs were driven by detailed downscaled climate data while others used coarser-resolution climate information as input). As common climate database EURO—CORDEX climate data was harnessed for historical conditions as well as three RCP scenarios (RCP2.6, RCP4.5 and RCP8.5), each simulated with three global circulation models (MPI-M-MPI-ESM-LR, ICHEC-EC-EARTH, NCC—NorESM1-M, all downscaled with the SMHI-RCA4 RCM), resulting in 12 climate scenarios. The climate data was obtained in a 0.11° x 0.11° spatial resolution and daily temporal resolution from the Copernicus Climate Data Store. (https://cds.climate.copernicus.eu/cdsapp#!/dataset/projections-cordex-domains-single-levels?tab=overview). GCMs were selected to cover a broad gradient of temperature and precipitation conditions [23] representative for the current and future climatic conditions in Europe.

For the harmonized dataset, the goal was to obtain daily climate data from EURO—CORDEX for all simulations. To achieve this, the best matching EURO—CORDEX trajectory was assigned to each simulation and for each simulation year the daily data from the best matching year from that scenario was extracted. In more detail, information on the type of climate trajectory used for the simulation run (baseline run without climate change, SRES, or RCP scenario family) was obtained from the metadata of each simulation. Baseline conditions refer to historical time series, while SRES and RCP scenarios refer to simulations under future climate scenarios. The best matching EURO—CORDEX scenario was assigned to each simulation based on a comparison between slopes (i.e. temporal change) of temperature and precipitation used in the simulations with those calculated from EURO—CORDEX scenarios for the simulation location. The difference of the slope for temperature and precipitation was obtained and the overall difference was calculated as ∆*T* + 0.1* ∆P to choose the trajectory with the smallest overall difference. Whenever simulation metadata contained specific information on the GCM and/or RCP used for the climate forcing used in the simulation, this information was harnessed to limit selection options from the EURO—CORDEX database. For instance, if the climate trajectory of a simulation was run with an RCP2.6 scenario, the temperature and precipitation slopes were only compared with the three RCP2.6 scenarios contained in our EURO—CORDEX selection. The best matching trajectory was then adjusted to the mean temperature and precipitation level of the simulation. Specifically, the difference between mean temperatures of the simulation and climate scenario trajectory and the multiplicative difference for precipitation was added. This was necessary to make sure that the adjusted time series represented the climate used in the simulations, as they may differ due to the relatively coarse resolution of the gridded climate data. Next, a time series from the adjusted scenario data was constructed, matching the individual years to each year in the simulation. For this, an index combining the additive difference between mean annual temperatures and the multiplicative difference of annual precipitation (again with ∆*T* + 0.1* ∆P) was calculated for each pair of simulation data year and climate scenario year. To prevent multiple occurrences of the same year, one of the three best matching years was randomly sampled with replacement. Finally, the thus constructed daily climate time series data for all variables (temperature, precipitation, radiation and VPD) was stored along the simulation it represents.

#### 4.3.2 Soil

Mirroring the approach taken with climate data, the simulation metadata provided by the modelling groups was facilitated to derive a consistent and quantitative soil data set across all simulations. While some simulation metadata contained exact numbers on all relevant soil variables considered (i.e. soil depth, soil texture (sand, silt, clay percentage), water holding capacity (WHC) and available nitrogen), others provided more descriptive values for water and nutrient conditions, such as soil water or fertility ratings (see Table S3). To complete missing data and convert descriptive ratings to quantitative soil characteristics pan-European datasets on soil information were leveraged. For soil depth, soil texture and WHC gridded data (1 km resolution) from the European Soil Data Center (ESDAC [24]) was obtained. Soil fertility was approximated by means of plant-available nitrogen, considering that nitrogen is the most important macro-nutrient for forests in Europe at the continental scale. Since data on plant-available nitrogen (i.e., the annual flux of mineralized nitrogen) is not available for large scales, we used a statistical approach to estimate mineralization rates based on fertility and climate data and combined them with data on nitrogen stocks. Specifically, the SQ1 nutrient availability map from the harmonized European soil database [25] was reclassified from the four classes of soil fertility considered therein to values of 100, 65, 40 and 20 kg/ha/year of plant-available nitrogen. In a next step, from the Soilgrids dataset [26] the nitrogen pool (in kg/m^2^) across Europe based on relative nitrogen content (g/kg) and bulk density (kg/m^3^) for soil layers up to 30 cm soil depth were obtained. The reclassified soil fertility map was divided by the nitrogen pool layer to approximate a coarse pseudo-mineralization rate. This pseudo-mineralization rate was then used as a dependent variable to calibrate a generalized linear model (GLM) with mean annual temperature, annual precipitation, temperature seasonality (obtained from CHELSA [27]; we used CHELSA data instead of EURO—CORDEX to create a layer of 1 km spatial resolution, matching the other soil layers) and soil pH (from Soilgrids) as predictor variables (D^2^ = 0.64). The calibrated model was used to project pseudo-mineralization rates for continental Europe at 1 × 1 km resolution providing more consistent estimates based on local soil and climate conditions. Finally, the nitrogen pool was multiplied with the modeled pseudo-mineralization rate to get an approximation of plant-available nitrogen.

### 4.4 Coverage of simulated data

The coverage of the current climate and soil space of Europe's forests by simulation data compiled here cover was investigated using the harmonized data (see Fig. 2). The historical climate was categorized into ten stratified bins of mean annual temperature (< −2 °C, −2 – 2 °C, 2–4 °C, 4 – 6 °C, 6 – 8 °C, 8 – 10 °C, 10 – 12 °C, 12 – 14 °C, 14 – 18 °C, > 18 °C), seven stratified bins of annual precipitation sum (< 400 mm, 400 – 550 mm, 550 – 750 mm, 750 – 1000 mm, 1000 – 1400 mm, 1400 – 2000 mm, > 2000 mm) and seven stratified bins of temperature seasonality (calculated as standard deviation of monthly mean temperature* 100, binned to 〈 200, 200 – 400, 400 – 500, 500 – 600, 600 – 700, 700 – 900, 〉 900). Simulations were assigned to their bins based on the climate grid cell (0.11°) of the simulation's location, and the area that is covered by the occupied bins was calculated. This approach likely underestimates the true climatic coverage of the simulations, as individual simulations (both stand-level and landscape-level) often encompass climatic gradients within a single cell. Soil conditions were categorized into five unique combinations of stratified values along the dimensions of soil texture (six classes from fine to coarse calculated with sand, silt and clay content), water holding capacity (<50 mm, 50 – 100 mm, 100 – 150 mm, 150 – 180 mm, >180 mm) and soil fertility (plant-available nitrogen of < 20, 20 – 40, 40 – 65, 65 – 100, >100 kg/ha/year). Again, each simulated location was located in the three-dimensional space of soil conditions to assess how well the compiled simulation data represented the soil conditions of Europe's forests.

## Limitations

The majority of simulations were sourced from iLand model outputs (73.2%, as shown in Table 2), totaling over 800,000 entries stored in large tables, which were unwieldy to manage. To mitigate this, we divided the simulation data into smaller chunks with different unique identifiers. Furthermore, the imbalance in the number of simulations from the various models creates a bias that needs consideration during database utilization for analytical purposes.

## Ethics Statement

We confirm to have read the ethical requirements for publication in Data in Brief and confirm that the current work does not involve human subjects, animal experiments, or any data collected from social media platforms.

## Declaration of Generative AI and AI-Assisted Technologies in the Writing Process

During the preparation of this work the authors used ChatGPT in order to improve readability. After using this tool, the authors reviewed and edited the content as needed and take full responsibility for the content of the publication.

## CRediT authorship contribution statement

**Marc Grünig:** Conceptualization, Methodology, Software, Formal analysis, Data curation, Writing – original draft, Writing – review & editing, Visualization. **Werner Rammer:** Conceptualization, Methodology, Software, Formal analysis, Data curation, Writing – original draft, Writing – review & editing, Funding acquisition. **Katharina Albrich:** Data curation, Writing – review & editing. **Frédéric André:** Data curation, Writing – review & editing. **Andrey L.D. Augustynczik:** Data curation, Writing – review & editing. **Friedrich Bohn:** Data curation, Writing – review & editing. **Meike Bouwman:** Data curation, Writing – review & editing. **Harald Bugmann:** Data curation, Writing – review & editing. **Alessio Collalti:** Data curation, Writing – review & editing. **Irina Cristal:** Data curation, Writing – review & editing. **Daniela Dalmonech:** Data curation, Writing – review & editing. **Miquel De Caceres:** Data curation, Writing – review & editing. **Francois De Coligny:** Data curation, Writing – review & editing. **Laura Dobor:** Data curation, Writing – review & editing. **Christina Dollinger:** Data curation, Writing – review & editing. **David I. Forrester:** Data curation, Writing – review & editing. **Jordi Garcia-Gonzalo:** Data curation, Writing – review & editing. **José Ramón González:** Data curation, Writing – review & editing. **Ulrike Hiltner:** Data curation, Writing – review & editing. **Tomáš Hlásny:** Data curation, Writing – review & editing. **Juha Honkaniemi:** Data curation, Writing – review & editing. **Nica Huber:** Data curation, Writing – review & editing. **Mathieu Jonard:** Data curation, Writing – review & editing. **Anna Maria Jönsson:** Data curation, Writing – review & editing. **Fredrik Lagergren:** Data curation, Writing – review & editing. **Mats Nieberg:** Data curation, Writing – review & editing. **Marco Mina:** Data curation, Writing – review & editing. **Frits Mohren:** Data curation, Writing – review & editing. **Christine Moos:** Data curation, Writing – review & editing. **Xaxier Morin:** Data curation, Writing – review & editing. **Bart Muys:** Data curation, Writing – review & editing. **Mikko Peltoniemi:** Data curation, Writing – review & editing. **Christopher PO Reyer:** Data curation, Writing – review & editing. **Ilié Storms:** Data curation, Writing – review & editing. **Dominik Thom:** Data curation, Writing – review & editing. **Maude Toïgo:** Data curation, Writing – review & editing. **Rupert Seidl:** Conceptualization, Methodology, Writing – original draft, Writing – review & editing, Funding acquisition.

## Data Availability

Data for: A harmonized database of European forest simulations under climate change (Original data) (Zenodo)

## Appendix Supplementary materials

Image, application 1
