# Supplementary material for: A harmonized database of European forest simulations under climate change
Source: Data Brief. 2024 Apr 3;54:110384. doi: 10.1016/j.dib.2024.110384 (PMC11033166; doi:10.1016/j.dib.2024.110384)
Supplement: Supplementary file 1 [file mmc1.docx]

**Supplementary Material**

**Supplementary tables**

*Table S1: Structure of the raw forest simulation data as provided by the individual modelling teams. Each row in this table describes a column in the simulation database.*

| **Section** | **Column Name** | **Unit** | **Description** |
| --- | --- | --- | --- |
| **General** | SourceID |  | ID to identify the source of the data (i.e. contributor). |
|  | SimulationID |  | Unique numeric identifier to distinguish multiple simulations run with the same driver data. The value was freely chosen by the contributors, and corresponds with the provided meta data (see below). |
|  | Year |  | Year of the simulation starting with 1 or the calendar year (e.g., 2000). |
| **Species composition** | Species1 | Code | Indicates the four-digit alphanumeric species identification key for a tree species, consisting of the first two letters of the scientific genus and species names, respectively (e.g., “piab” for *Picea abies*). |
|  | Proportion1 | % | The proportion of the species on total stands basal area in per cent. Species and corresponding proportions were ordered by proportions and cut off at five species. |
|  | Species2 | Code |  |
|  | Proportion2 | % |  |
|  | Species3 | Code |  |
|  | Proportion3 | % |  |
|  | Species4 | Code |  |
|  | Proportion4 | % |  |
|  | Species5 | Code |  |
|  | Proportion5 | % |  |
| **Structure** | MeanHeight | m | Mean height in meters |
|  | MaxHeight and MinHeight | m | Maximum height and minimum height in meters (applicable if mean height was not provided) |
| **Functioning** | LAI | m²/m² | Leaf Area Index (one-sided or projected) |
| **Climate** | Temp | °C | Mean annual temperature of the year in °C |
|  | Precip | mm | Amount of precipitation in mm of the simulation year |

*Table S2: Structure of the metadata, provided by the individual modeling teams for each simulation batch.*

| *Section* | *Column name* | *Description* |
| --- | --- | --- |
| General | SourceID | ID to identify the source of the data (i.e. contributor). |
|  | SimulationID | Unique numeric identifier of the simulation created by the contributor (links to the SimulationID in the data table, Table S1). SimulationID are unique in this table. |
|  | Model | Name of the forest model used to run the simulation |
|  | ModelDOI | Most suitable scientific reference for the model, expressed as digital object identifier (DOI) |
|  | Lon | Location of the plot - X-coordinate (WGS84, EPSG:4326) |
|  | Lat | Location of the plot - Y-coordinate (WGS84, EPSG:4326) |
|  | Country | Country name (used for sanity check of coordinates) |
|  | DOI | DOI if the simulation is from a published paper |
| Soil water | WHC | Estimated water holding capacity of the site (mm) |
|  | TextureSand | % sand content of the soil |
|  | TextureSilt | % silt content of the soil |
|  | TextureClay | % clay content of the soil |
|  | SoilDepth | Rock-free (> 2mm diameter) soil depth that can be accessed by plant roots |
|  | SoilWaterRating | Relative rating of the soil water availability between 0 (very low) and 1 (excellent). (see Table S1). Only applicable if exact values for soil water information were not available |
| Nutrients | AvailableNitrogen | Plant available nitrogen (kg/ha/yr) |
|  | FertilityRating | Relative rating of soil fertility between 0 (very low) and 1 (excellent). Only applicable if exact values for nitrogen availability were not available. (see Table S3) |
| Climate | Climate | Classification of the climate scenario family. (selected from options in Table S4) |
|  | GCM | Full name of global circulation model (GCM) (e.g., MPI-M-MPI-ESM-MR) |
|  | RCM | Full name of regional climate model (RCM) (e.g., CLMcom-KIT-CCLM5-0-15) |
| Management | Management | ‘N’ no management, ‘B’ business as usual. |

*Table S3: Examples for the descriptive ratings used to describe soil water availability and soil fertility.*

| **Soil conditions** | **Rating** |
| --- | --- |
| Very shallow, sandy soils, hardly allow any vegetation (WHC ~30mm) | 0 |
| Average soil with average texture and soil depth (WHC ~ 100mm) | 0.5 |
| Excellent water storage capacity and water supply, deep soils with predominately loamy texture (WHC ~ 200mm) | 1 |

| **Soil conditions** | **Rating** |
| --- | --- |
| Very low nutrient supply, allows only limited growth of least demanding pioneer species (plant available nitrogen <= 20kg/ha/yr), FAO Rating=4 | 0 |
| Low nutrient supply, growth is severely limited (~40 kg/ha/yr), FAO Rating=3 | 0.33 |
| Moderate nutrient supply (~65kg/ha/yr), FAO Rating=2 | 0.66 |
| Excellent nutrient supply with no growth limitation (Available Nitrogen >= 100kg/ha/yr), FAO Rating=1 | 1 |

*Table S4: Information on climate scenario family as reported by the modeling teams.*

| **Climate scenario family (“Climate”)** |
| --- |
| Baseline (continuation of historic climate condition, no climate change) |
| SRES A1 |
| SRES A2 |
| SRES B1 |
| SRES B2 |
| RCP2.6 |
| RCP4.5 |
| RCP6.0 |
| RCP8.5 |
| Observed (from meteorological stations) |
| Other |
